# Identification of quantitative trait loci (QTLs) and candidate genes for seed shape and 100-seed weight in soybean [*Glycine max* (L.) Merr.]

**DOI:** 10.3389/fpls.2022.1074245

**Published:** 2023-01-04

**Authors:** Rahul Kumar, Manisha Saini, Meniari Taku, Pulak Debbarma, Rohit Kumar Mahto, Ayyagari Ramlal, Deepshikha Sharma, Ambika Rajendran, Renu Pandey, Kishor Gaikwad, S. K. Lal, Akshay Talukdar

**Affiliations:** ^1^ Division of Genetics, Indian Council of Agricultural Research (ICAR)-Indian Agricultural Research Institute (IARI), New Delhi, India; ^2^ School of Biotechnology, Institute of Science, Banaras Hindu University (BHU), Varanasi, Uttar Pradesh, India; ^3^ Division of Plant Physiology, Indian Council of Agricultural Research (ICAR)-Indian Agricultural Research Institute, New Delhi, India; ^4^ Division of Molecular Biology and Biotechnology, Indian Council of Agricultural Research (ICAR)- National Institute for Plant Biotechnology, New Delhi, India

**Keywords:** soybean, seed shape, seed weight, QTL, candidate genes, marker assisted breeding

## Abstract

Seed size and shape are important traits determining yield and quality in soybean. Seed size and shape are also desirable for specialty soy foods like tofu, natto, miso, and edamame. In order to find stable quantitative trait loci (QTLs) and candidate genes for seed shape and 100-seed weight, the current study used vegetable type and seed soybean-derived F_2_ and F_2:3_ mapping populations. A total of 42 QTLs were mapped, which were dispersed across 13 chromosomes. Of these, seven were determined to be stable QTLs and five of them were major QTLs, namely *qSL-10-1, qSW-4-1, qSV-4-1, qSLW-10-1*, and *qSLH-10-1*. Thirteen of the 42 QTLs detected in the current study were found at known loci, while the remaining 29 were discovered for the first time. Out of these 29 novel QTLs, 17 were major QTLs. Based on Protein Analysis Through Evolutionary Relationships (PANTHER), gene annotation information, and literature search, 66 genes within seven stable QTLs were predicted to be possible candidate genes that might regulate seed shape and seed weight in soybean. The current study identified the key candidate genes and quantitative trait loci (QTLs) controlling soybean seed shape and weight, and these results will be very helpful in marker-assisted breeding for developing soybean varieties with improved seed weight and desired seed shape.

## 1 Introduction

Soybean [*Glycine max* (L.) Merr.] is one of the most economically important crops in the world, which is also used as a model plant for research on legumes (Kumar et al., 2022). It is a rich source of both edible oil and plant-based protein, which also fixes atmospheric nitrogen through a symbiotic interaction with soil microorganisms (Wang et al., 2019). Soybean is a marvellous legume meeting daily oil and protein dietary needs (Rajendran et al., 2022). Soybean is widely grown and consumed globally and constitutes nearly 28% of vegetable oil and 70% of protein meals worldwide (SoyStats, 2021). Because of its great nutritional content, it is used in both human food and animal feed (Ramlal et al., 2022a; 2022b). Besides, it is used in the pharmaceutical and cosmetic industries. The seed size and color are important for specific uses of soybean seeds. For example, in specialty soy food products, such as tofu, natto, miso, and edamame, seed weight and seed shape are critically considered (Cui et al., 2004). For food-type soybean, round seeds are preferred over others. Similarly, large seeds are considered to be ideal for tofu, edamame, and miso production, while small seeds are for natto manufacture (Wilson, 1995; Yan et al., 2017).

Despite being one of the most economically significant leguminous seed crops and producing more than a quarter of the world’s edible oil and animal feed, soybean has a rather low yield level (Egli, 2008). The seed size and shape play a key role in determining seed weight and yield in soybean (Salas et al., 2006; Yan et al., 2017). Seed appearance including seed length (SL), seed width (SW), and seed height (SH), as well as seed shape traits such as length-to-width (SLW), length-to-height (SLH), and width-to-height (SWH) ratios, affect seed yield (Xu et al., 2011). Seed size, which is measured as 100-seed weight (100-SW), is a fitness trait that is essential for environmental adaptation (Tao et al., 2017). Under natural conditions, greater seed resources stored in larger seeds enable seedlings to grow more rapidly at the seedling stage and increase competitiveness and survival (Manga and Yadav, 1995). Increased seed number also translates directly into fitness, resulting in selection pressure to produce more (and thus smaller) seeds (Westoby et al., 1992). Environmental factors can also exert a strong influence on seed size by affecting assimilate supply (Borrell et al., 2014) and carbon translocation (Zolkevich et al., 1958). However, such traits are very complex in nature and difficult to improve through normal breeding approaches.

An effective method to clarify complicated trait architecture is quantitative trait loci (QTL) analysis. In soybean, natural selection for larger seeds has led to an accumulation of minor QTLs, and QTL mapping has shed light on these evolutionary changes (Salas et al., 2006; Hina et al., 2020). More than 400 QTLs for seed size and shape are now documented in the USDA Soybean Genome Database (SoyBase; http://www.soybase.org), however, majority of these QTLs are unconfirmed i.e. these are neither mapped across several environments nor validated on mapping population with different genetic background (Hina et al., 2020). For instance, 16 QTLs for seed size and shape were found on 12 different soybean chromosomes by Mian et al. (1996). On 16 soybean chromosomes, Hoeck et al. (2003) found 27 QTLs related to seed size, while Li et al. (2008) found three QTLs for seed length (SL) on Chr. 7, Chr.13, and Chr.16. Lü et al. (2011) found 19 main-effect QTLs (M-QTLs) and three epistatic-effect QTLs (E-QTLs) for SL on eight chromosomes. In the recombinant inbred line (RIL) population obtained from a hybrid between Li-shui-zhong-zi-huang and Nannong493-1, Xie et al. (2014) finely mapped QTLs for soybean seed size traits on Chr.6. For six soybean seed size and shape variables, Hina et al. (2020) found 88 main and epistatic-effect QTLs. Similarly, 42 additive effect QTLs for seed traits were mapped by Li et al. (2020). However, across various genetic backgrounds and conditions, only a few numbers of stable QTLs associated with seed and yield-related traits [seed length (SL), seed width (SW), seed height (SH), length-to-width (SLW), length-to-height (SLH), and width-to-height (SWH) and 100-seed weight (100-SW)] have been found. Therefore, for effective employment of QTLs in marker-assisted breeding, it is essential to find QTLs and confirm them in a variety of backgrounds and conditions. Despite this, there are only a few papers focusing on the mapping of QTLs for seed size and shape using the high-density map in various genetic backgrounds of soybean (Karikari et al., 2019). In addition, most of the published reports did not mine the candidate genes for seed shape and seed weight (Zhang et al., 2004; Niu et al., 2013; Kato et al., 2014; Wu et al., 2018). Keeping these issues in mind, the current study was attempted using an F_2_ and F_2:3_ population derived from a cross between vegetable type (AGS 457) and seed types (SKAF 148) soybean to determine the most significant genomic areas and potential genes for seed size and shape in soybeans. These findings will be useful for marker-assisted breeding (MAB) for the seed size and shape of soybean seeds and create soybean varieties with increased yield and quality.

## 2 Materials and methods

### 2.1 Plant materials

In this study, F_2_ and F_2:3_ populations were used. These populations were developed by crossing soybean genotypes AGS 457 and SKAF 148. The SKAF 148 is a small-seeded grain type soybean while AGS 457 is a large-seeded vegetable soybean genotype. Both genotypes varied widely in seed size and shape. Young leaves were collected from 237 F_2_ plants and the two parental genotypes that were grown in the field of the Indian Agricultural Research Institute in New Delhi, India, during the 2020 growing season (July 2020-November 2020). Furthermore, 237 F_2:3_ lines and the two parents were sown in a field of Indian Agricultural Research Institute, New Delhi, India in a randomized complete block design with two replications in the 2021 growing seasons. Each plot has one row of 1 m long constituting 10 plants in each row with a space of 10 cm between the adjacent plants. Five individuals from each of the F_2:3_ families were taken for phenotypic evaluation.

### 2.2 Trait measurement

**Figure 1:**
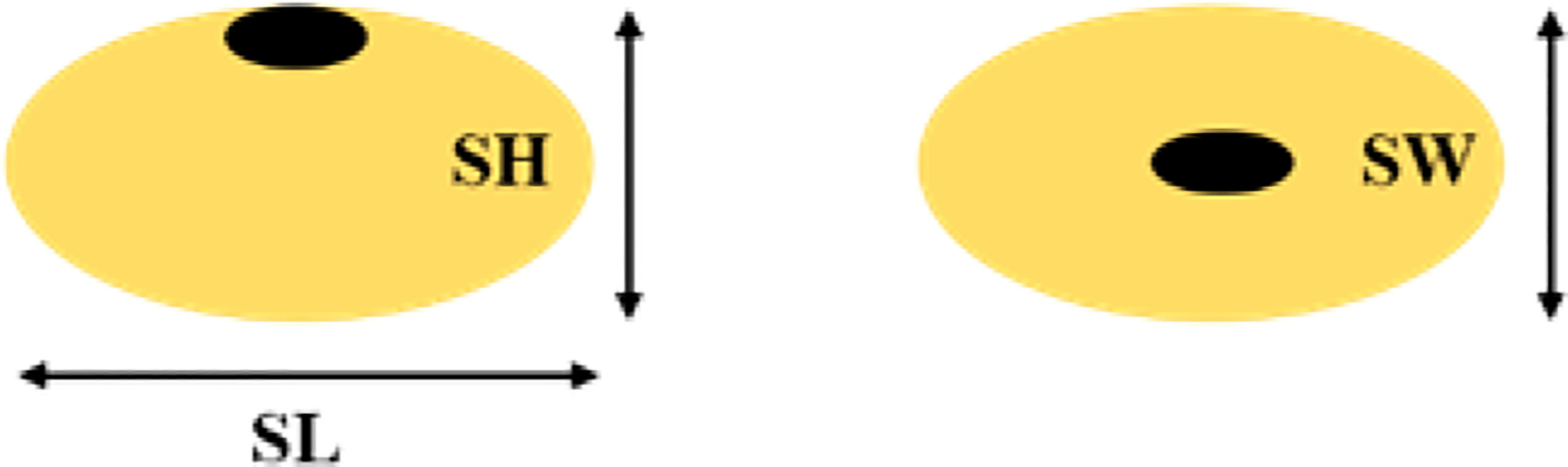
Seed Length (SL), Seed Width (SW) and Seed Height (SH).

The traits were assessed in the plants of F_2_ and F_2:3_ mapping populations. Seed shape and 100 seed weight were evaluated in both generations. Using an electronic Vernier Caliper, the following seed dimensions were measured: length (mm), width (mm), and height (mm) (**Figure 1**). This information was used to calculate the seed’s volume (mm^3^) as width × height × length, seed length to width ratio (SLW), seed length to height ratio (SLH), and seed width to height ratio (SWH) (Salas et al., 2006). A weighing machine (KERN ABT 320-4M) was used to calculate the weight (g) of 100 seeds (100 seed weight). The Pearson phenotypic correlation coefficients between traits were calculated using R. (R Core Team, 2013).

### 2.3 DNA extraction and SSR genotyping

Genomic DNA was extracted from the young leaves using a modified CTAB method as described by Lodhi et al. (1994). On an ethidium bromide-stained 0.8% agarose gel, the DNA’s purity was examined. Based on the consensus soybean genetic linkage map published by Cregan et al. (1999) and Song et al. (2004), SSR markers scattered throughout the 20 genetic linkage groups were chosen. A set of 638 SSR markers were used for polymorphic survey and 123 polymorphic markers were employed for genotyping of the F_2_ population.

### 2.4 Map construction and QTL detection

We used the program QTL IciMapping V4.2 to make the linkage map and find QTLs. A genetic distance of 50 cM and a minimum LOD score of 2.5 was used to construct the linkage map connecting the markers. Kosambi’s mapping function (Kosambi, 1944) was used to calculate map distances. Utilizing the composite interval mapping approach (CIM), the QTL analysis was performed (Zeng, 1994). A LOD score of 2.5 was maintained to confirm the presence of a QTL in a particular genomic region.

### 2.5 Candidate gene prediction analysis

In this study, when a particular QTL was mapped in both the F_2_ and the F_2:3_ generations, it was considered a stable QTL. Model genes within the genomic physical position of the stable QTLs on the soybean genome (Glyma2.0) available at *SoyBase* (http://www.soybase.org) and *EnsemblPlants* (https://plants.ensembl.org) were downloaded. Phytozome 13 (http://phytozome-next.jgi.doe.gov) was used to conduct a Gene Ontology (GO) enrichment analysis for all the genes in each QTL region. In order to facilitate high-throughput analysis according to family and subfamily, molecular function, biological process, and pathway, the projected candidate genes were then subjected to Protein Analysis Through Evolutionary Relationships (PANTHER) Classification System.

## 3 Results

### 3.1 Phenotypic variation in parents and the population

**Figure 2:**
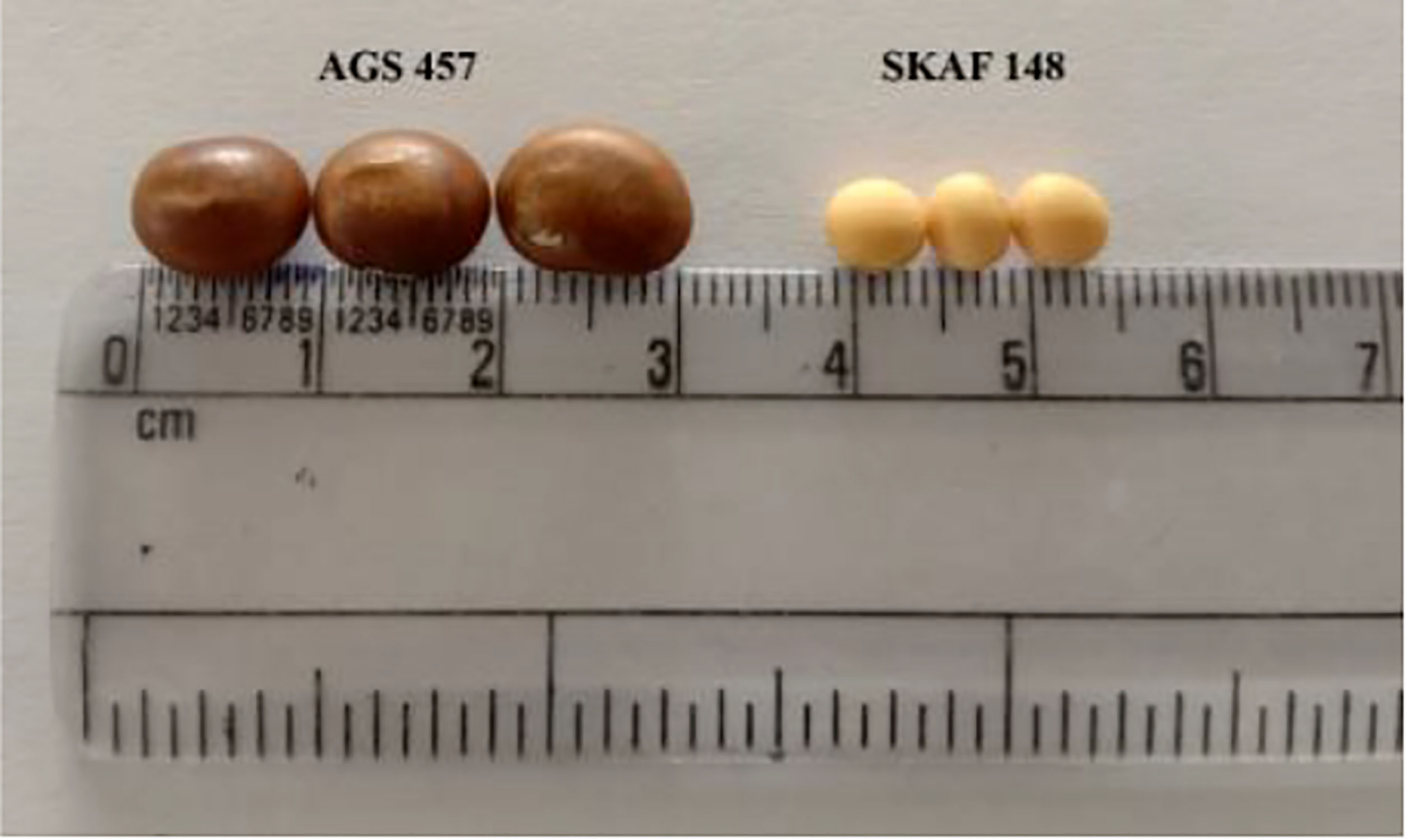
Comparison of the seed size and seed shape between the AGS 457 and SKAF 148.

**Figure 3:**
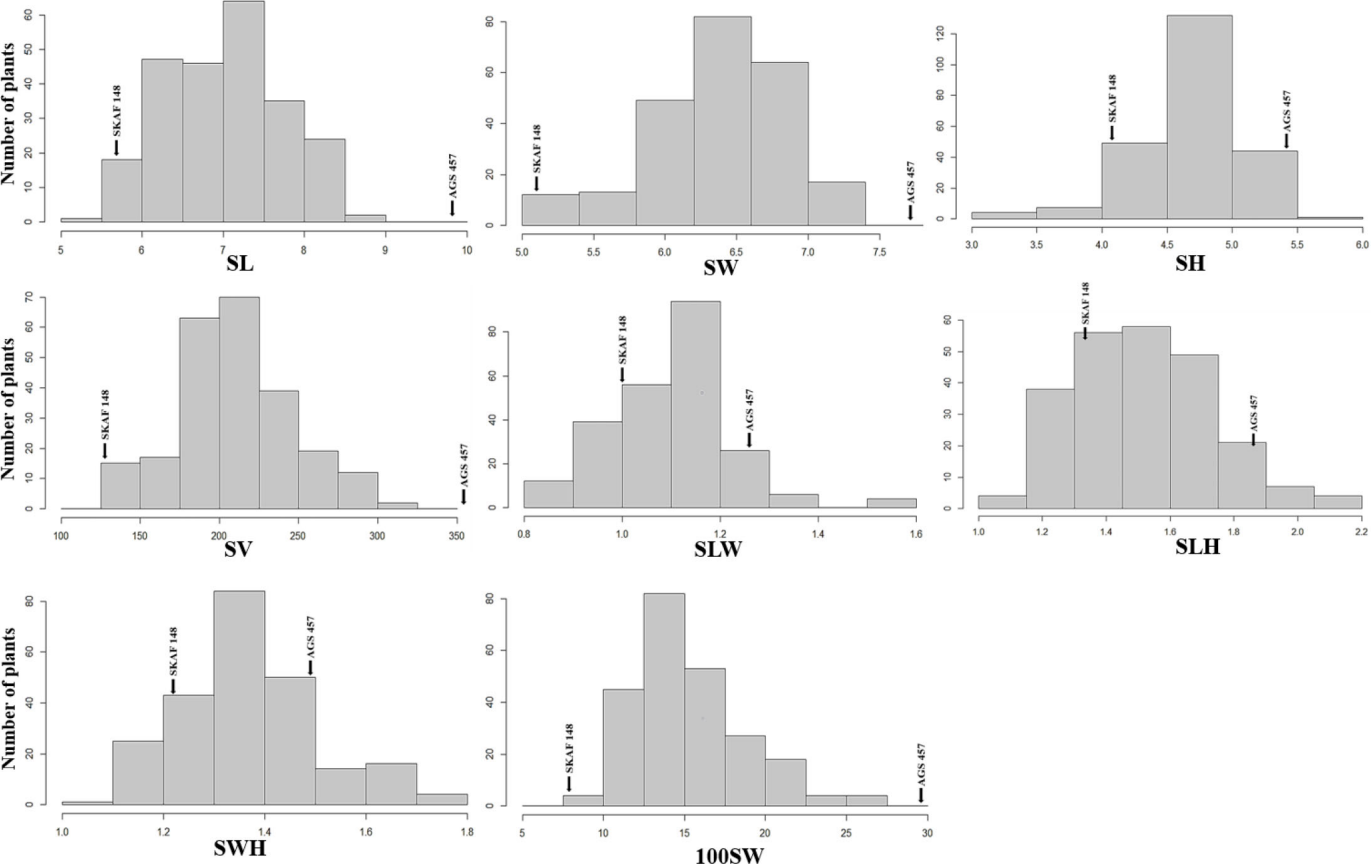
Frequency distributions of seed-related traits [SL, Seed Length (mm); SW, Seed Width (mm); SH, Seed Height (mm); SV, Seed Volume (mm^3^); SLW, Seed Length to Width ratio; SLH, Seed length to Height ratio; SWH, Seed Width to Height ratio and 100SW, 100 seed weight (g)] in the F_2:3_ population derived from the cross of AGS 457 X SKAF 148. Parental means are denoted with arrows.

There was a considerable variation in seed sizes and other seed features between the parental genotypes AGS 457 and SKAF 148 (**Table 1**) (**Figure 2**). The frequency distribution of the F_2_ data exhibited that all the seed-related traits were distributed normally (**Figure 3**), indicating their polygenic inheritance. Transgressive segregation was also noted for all the traits studied. In the F_2:3_ population, significant correlation coefficients for seed traits ranged from -0.135 to 0.75. (**Table 2**). Seed Width (SW), Seed Volume (SV), Seed Length to Width Ratio (SLW), Seed Length to Height Ratio (SLH), Seed Width to Height Ratio (SWH), and 100 Seed Weight (100SW) all had positive associations with seed length (SL). However, there was no discernible relationship between seed length and seed height. Seed length, Seed width, and Seed Volume were found significantly correlated with 100 seed weight while seed height had a non-significant correlation with 100 seed weight. Seed Length and Width ratio (SLW) had a significant positive association with seed volume while SLH and SWH had a non-significant association with seed volume that indicates seed length and seed width as the major determinant of seed shape and seed weight of soybean.

**Table 1:** Descriptive statistics of evaluated quantitative traits in parents, F_2_ population and F_2:3_ population.

Traits	Parents		Difference	F_2_ Population		F_2:3_ Population	
	AGS 457	SKAF 148	Between Parents	Range	Mean ± SD	Range	Mean ± SD
SL	9.82	5.73	4.09	5.67-8.61	7.11 ± 0.70	5.45-8.66	7.01 ± 0.72
SW	7.85	5.24	2.61	4.9-7.59	6.31 ± 0.58	5.03-7.37	6.39 ± 0.47
SH	5.35	4.24	1.11	2.4-5.63	4.76 ± 0.62	3.41-5.55	4.68 ± 0.40
SV	412.42	127.31	285.11	61.1-338.15	220.33 ± 58.22	130.9-302.37	210.51 ± 36.10
SLW	1.25	1.09	0.16	0.85-1.48	1.13 ± 0.10	0.83-1.59	1.10 ± 0.13
SLH	1.84	1.35	0.49	1.05-2.47	1.5 ± 0.20	1.13-2.08	1.51 ± 0.21
SWH	1.47	1.24	0.23	1.09-2.16	1.35 ± 0.15	1.08-1.75	1.37 ± 0.14
100SW	29.68	8.61	21.07	6.48-24.0	13.49 ± 3.82	9.17-25.44	15.31 ± 3.46

SL, Seed Length (mm); SW, Seed Width (mm); SH, Seed Height (mm); SV, Seed Volume (mm3); SLW, Seed Length-to- Width ratio; SLH, Seed length-to-Height ratio; SWH, Seed Width-to-Height ratio; 100SW, 100 Seed Weight (g).

**Table 2:** Pearson phenotypic correlation coefficient among seed-related traits in F_2:3_ population.

	SL	SW	SH	SV	SLW	SLH	SWH
SL	1						
SW	0.216**	1					
SH	-0.050NS	0.282**	1				
SV	0.678**	0.692**	0.581**	1			
SLW	0.750**	-0.470**	-0.242**	0.139*	1		
SLH	0.749**	-0.030NS	-0.691**	0.105NS	0.695**	1	
SWH	0.193**	0.476**	-0.703**	-0.028NS	-0.135*	0.607**	1
100SW	0.235**	0.187**	0.111NS	0.279**	0.094NS	0.084NS	0.031NS

*, Significant at 5% level of significance; **, Significant at 1% level of significance; NS, Non-significant.

### 3.2 Linkage map construction

The two parents were examined for genetic polymorphism using 638 SSR markers, out of which, 123 markers (19.28%) were found to be polymorphic. The polymorphic markers were selected to genotype the plants in the F_2_ generation. The linkage map constructed with these markers developed twenty separate linkage groups (LGs), which had a total length of 2005.7cm covering 79.5% of the entire soybean genome (2523.6cM). The majority of the markers comprising the linkage groups had map locations, which corresponded well with the soybean composite map (http://soybase.agron.iastate.edu/); however, minor variation in the map distance has been observed among the markers in the map.

### 3.3 QTL mapping and analysis

**Figure 4:**
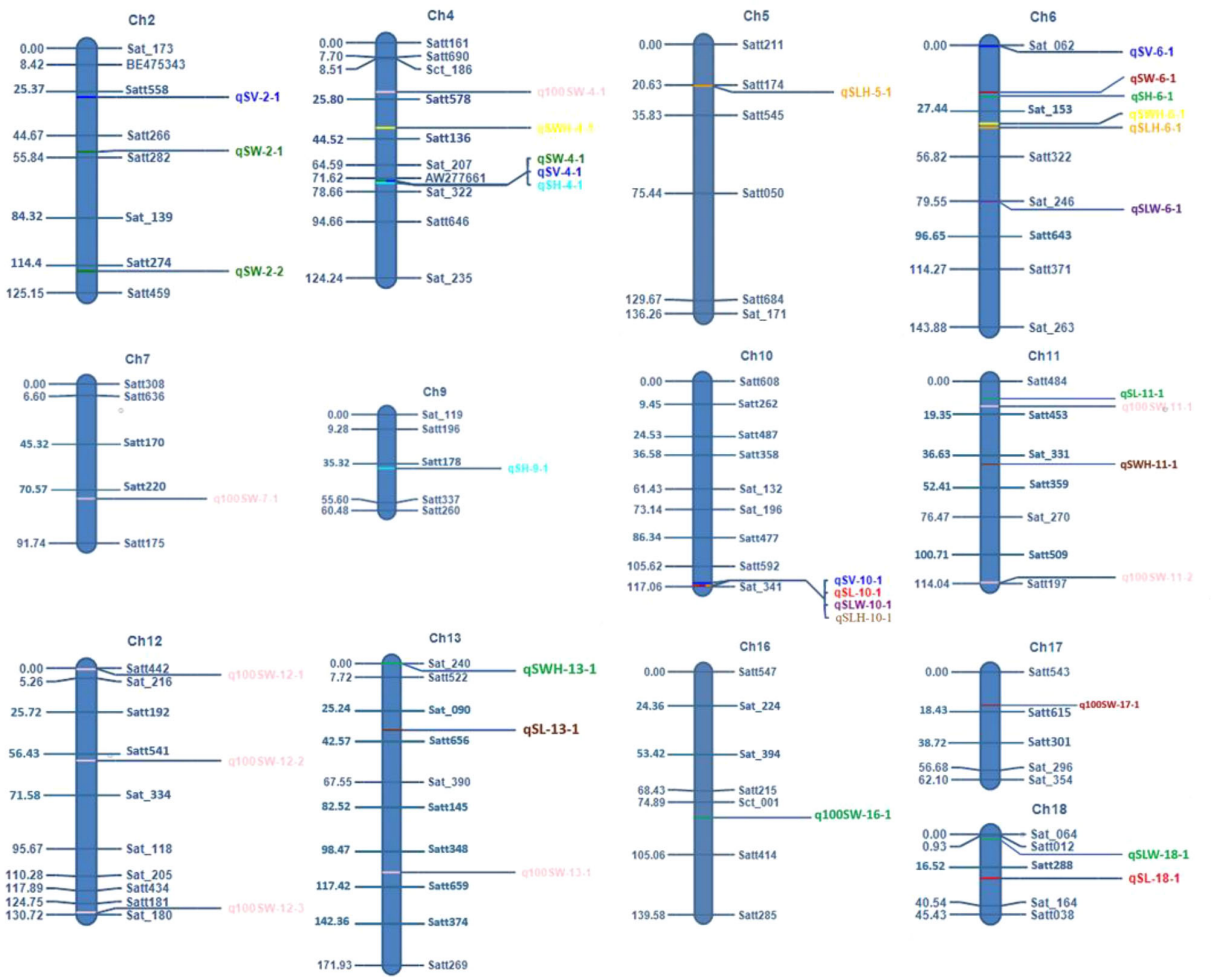
Mapping positions of various seed shape and 100 seed weight QTLs in this study.

The F_2_ and F_2:3_ populations developed in this study were used to map and analyze the QTLs for the seed-related traits in soybean. In the F_2_ population, 17 unique QTLs were mapped using Composite Interval Mapping (CIM), with each QTL accounting for 5.03% to 23.79% of the phenotypic variances of the corresponding traits. The 17 QTLs were distributed on eight chromosomes, viz., Chrs.4, 6, 10, 11, 13, 16, 17, and 18 (**Figure 4**). There were one or more QTLs on each of these eight chromosomes, with Chr.06 having the highest number i.e., four QTLs. In the F_2:3_ population, 25 QTLs were identified and mapped for the seed-related traits. The phenotypic variance explained (PVE) by any individual QTL ranged from 3.8% to 33.94%. The detected QTLs were discovered across eleven distinct chromosomes viz., Chromosome 2, 4, 5, 6, 7, 9, 10, 11, 12, 13, and 18 (**Figure 4**). The maximum number of QTLs i.e., 5 were found on chromosome 4.

For seed length, three QTLs viz., *qSL-10-1, qSL-11-1*, and *qSL-13-1* were mapped on chromosome 10, 11, and 13, in the F_2_ generation plants, while two QTLs *qSL-10-1* and *qSL-18-1* were mapped in the F_2:3_ population on chromosome 10 and 18 respectively. The first major QTL *qSL-10-1* on chromosome 10 was mapped in the marker interval Satt592-Sat_341 with a LOD score of 4.71 and a PVE of 13.7%. On chromosome 13, the second major QTL, *qSL-13-1* was mapped in the Sat_090-Satt656 interval and had a LOD score of 3.18, and accounted for 22.84% of the phenotypic variations of seed length. On chromosome 18, the third major QTL, *qSL18-1*, was located in the marker region Satt288-Sat_164 Sattwith a LOD score of 2.87, accounting for 17.21% of PVE. Since the QTL *qSL-10-1* was discovered in both the F_2_ and F_2:3_ populations, it was regarded as a stable QTL (**Table 3**). The allele contributed by the genotype SKAF 148 reduced the seed length in both the mapping populations

Four significant QTLs for seed width, designated as *qSW-2-1, qSW-2-2, qSW-4-1*, and *qSW-6-1*, were found on chromosomes 2, 4, and 6. The QTL *qSW-4-1* was found to be a major effect stable QTL and was mapped in both the mapping populations (**Table 3**). It was mapped in the marker interval AW277661-Sat_322 on chromosome 4 with LOD score of 7.58 with a PVE value of 14.15%. The alleles from the genotype AGS 457 contributed positively to the seed width. For seed height, one QTL i.e., *qSH-6-1* was mapped on F_2_ plants (**Table 4**) while two more QTLs viz., *qSH-4-1* and *qSH-9-1* were mapped on F_2:3_ populations (**Table 5**). Out of these, only two QTLs viz., *qSH-4-1* and *qSH-9-1* were major ones with PVE 17.52% and 17.28%, respectively. There was no stable QTLs found for the seed height. Similarly, for seed volume, two QTLs viz., QTLs *qSV-2-1* and *qSV-4-1* were mapped on the F_2_ population (**Table 4**) while four QTLs viz., QTLs *qSV-2-1, qSV-4-1*, *qSV-6-1* and *qSV-10-1* were mapped in the F_2:3_ population (**Table 5**). The QTLs viz., *qSV-4-1* and *qSV-6-1* appeared to be consistent QTL with higher PVE (**Table 3**). The alleles from SKAF148 were found to reduce the seed volume.

**Table 3:** Consistent QTLs for yield-related traits discovered in both F_2_ and F_2:3_ mapping populations derived from AGS 457 X SKAF 148.

Trait	QTL	Chr. No. (LG)	Marker Interval	Map Pos. (cM)	LOD
Seed Length (mm)	*qSL-10-1*	10 (O)	Satt592-Sat_341	117	4.71
Seed Width (mm)	*qSW-4-1*	4 (C1)	AW277661-Sat_322	73	7.58
Seed Volume (mm3)	*qSV-4-1*	4 (C1)	AW277661-Sat_322	73	12.34
	*qSV-6-1*	6 (C2)	Sat_062-Sat_153	0	3.47
Seed Length to Width Ratio	*qSLW-10-1*	10 (O)	Satt592-Sat_341	117	2.83
Seed Length to Height Ratio	*qSLH-10-1*	10 (O)	Satt592-Sat_341	117	4.92
100 Seed weight (g)	*q100SW-11-1*	11 (B1)	Satt484-Satt453	15	4.12

**Table 4:** QTLs identified for seed-related traits in the F_2_ mapping population derived from AGS 457 X SKAF 148.

Trait	QTL	Chr. No. (LG)	Marker Interval	Map Pos. (cM)	LOD	PVE (%)	Add.
Seed Length (mm)	*qSL-10-1*	10(O)	Satt592-Sat_341	117	3.70	5.93	0.35
	*qSL-11-1*	11 (B1)	Satt484-Satt453	11	2.92	8.87	-0.17
	*qSL-13-1*	13 (F)	Sat_090-Satt656	37	3.18	22.84	0.94
Seed Width (mm)	*qSW-4-1*	4 (C1)	AW277661-Sat_322	73	2.66	6.61	0.23
	*qSW-6-1*	6 (C2)	Sat_062-Sat_153	19	3.12	17.56	0.37
Seed Height (mm)	*qSH-6-1*	6 (C2)	Sat_062-Sat_153	23	2.95	9.24	0.54
Seed Volume (mm3)	*qSV-4-1*	4 (C1)	AW277661-Sat_322	73	2.66	5.65	27.03
	*qSV-6-1*	6 (C2)	Sat_062-Sat_153	0	2.60	15.64	29.25
Seed Length to Width Ratio	*qSLW-6-1*	6 (C2)	Satt322-Sat_246	79	3.31	10.95	-0.03
	*qSLW-10-1*	10 (O)	Satt592-Sat_341	117	6.97	20.66	0.11
	*qSLW-18-1*	18 (G)	Satt012-Satt288	3	3.13	6.89	0.06
Seed Length to Height Ratio	*qSLH-10-1*	10(O)	Satt592-Sat_341	117	2.55	12.67	0.13
Seed Width to Height Ratio	*qSWH-11-1*	11 (B1)	Sat_331-Satt359	42	4.12	18.27	-0.32
	*qSWH-13-1*	13 (F)	Sat_240-Satt522	0	3.41	6.14	-0.07
100 Seed weight (g)	*q100SW-11-1*	11 (B1)	Satt484-Satt453	15	3.49	14.67	-0.84
	*q100SW-16-1*	16 (J)	Sct_001-Satt414	86	3.14	5.85	2.36
	*q100SW-17-1*	17 (D2)	Satt543-Satt615	16	3.37	14.37	0.27

**Table 5:** QTLs identified for yield-related traits in the F_2:3_ mapping population derived from AGS 457 X SKAF 148.

Trait	QTL	Chr. No. (LG)	Marker Interval	Map Pos. (cM)	LOD	PVE (%)	Add.
Seed Length (mm)	*qSL-10-1*	10 (O)	Satt592-Sat_341	117	4.71	13.70	0.56
	*qSL-18-1*	18 (G)	Satt288-Sat_164	23	2.87	17.21	-0.06
Seed Width (mm)	*qSW-2-1*	2 (D1b)	Satt266-Satt282	53	8.21	17.09	0.08
	*qSW-2-2*	2 (D1b)	Satt274-Satt459	116	3.78	12.85	0.12
	*qSW-4-1*	4 (C1)	AW277661-Sat_322	73	7.58	14.15	0.45
Seed Height (mm)	*qSH-4-1*	4 (C1)	AW277661-Sat_322	74	5.20	17.52	0.35
	*qSH-9-1*	9 (K)	Satt178-Satt337	37	3.13	17.28	-0.24
Seed Volume (mm^3^)	*qSV-2-1*	2 (D1b)	Satt558-Satt266	27	3.23	13.42	0.08
	*qSV-4-1*	4 (C1)	AW277661-Sat_322	73	12.34	33.94	43.59
	*qSV-6-1*	6 (C2)	Sat_062-Sat_153	0	3.17	6.78	2.68
	*qSV-10-1*	10 (O)	Satt592-Sat_341	115	2.96	6.16	18.10
Seed Length to Width Ratio	*qSLW-10-1*	10 (O)	Satt592-Sat_341	117	2.83	19.60	0.08
Seed Length to Height Ratio	*qSLH-5-1*	5 (A1)	Satt174-Satt545	21	2.57	7.52	0.08
	*qSLH-6-1*	6 (C2)	Sat_153-Satt322	38	3.32	18.23	0.18
	*qSLH-10-1*	10 (O)	Satt592-Sat_341	117	4.92	16.67	0.16
Seed Width to Height Ratio	*qSWH-4-1*	4 (C1)	Satt578-Satt136	41	3.21	12.21	-0.14
	*qSWH-6-1*	6 (C2)	Sat_153-Satt322	35	3.45	11.42	0.18
100 Seed weight (g)	*q100SW-4-1*	4 (C1)	Sct_186-Satt578	23	3.82	11.41	3.34
	*q100SW-7-1*	7 (M)	Satt220-Satt175	74	3.34	8.47	-2.65
	*q100SW-11-1*	11 (B1)	Satt484-Satt453	15	4.12	8.72	-2.19
	*q100SW-11-2*	11 (B1)	Satt509-Satt197	114	4.23	7.32	2.68
	*q100SW-12-1*	12 (H)	Satt442-Sat_216	0	3.74	3.80	-1.54
	*q100SW-12-2*	12 (H)	Satt541-Sat_334	58	3.37	9.74	-3.26
	*q100SW-12-3*	12 (H)	Satt181-Sat_180	130	2.61	4.82	-1.40
	*q100SW-13-1*	13 (F)	Satt348-Satt659	107	3.31	9.87	3.32

An important seed trait that affects seed shape is the seed length-to-width ratio. In F_2_ and F_2:3_ populations, a total of four QTLs were mapped for this trait. Out of these, two major effects QTL viz., *qSLW-6-1* and *qSLW-10-1* were located on chromosomes 6 and 10 with PVE 10.95% and 19.6%, respectively. The QTL *qSLW-10-1* was mapped in both generations and hence can be considered a stable one (**Table 3**). Similarly, four QTLs for the seed length-to-height ratio were identified. In the F_2_ population, one QTL *qSLH-10-1* on chromosome 10 was mapped (**Table 4**) while in the F_2:3_ population, three QTLs on chromosomes 5, 6, and 10 were mapped (**Table 5**). *qSLH-6-1* and *qSLH-10-1*, two significant QTLs, were located on chromosomes 6 and 10, respectively. The QTL *qSLH-10-1*, which appeared on both the populations in the marker interval Satt592-Sat_341, had 16.67% PVE and appeared to be a major QTL (**Table 3**). Similarly, for the seed width-to-height ratio, four QTLs viz., *qSWH-4-1, qSWH-6-1, qSWH-11-1* and *qSWH-13-1* were mapped; however, none were consistent QTL.

An important economic factor influencing soybean yield is seed weight. For 100 seed weight (100SW), eleven QTLs were mapped, of which three QTLs were mapped in the F_2_ population on chromosomes 11, 16, and 17 (**Table 4**) while eight QTLs were mapped in the F_2:3_ population on chromosomes 4, 7, 11, 12, and 13 (**Table 5**). Three major QTLs viz., *q100SW-4-1, q100SW-11-1*, and *q100SW-17-1* had PVE 11.41%, 14.67%, 14.37%, respectively. Mapped in both F_2_ and F_2:3_ populations, QTL *q100SW-11-1* at the marker interval Satt484-Satt453 which accounted for 14.67% of PVE could be regarded as a stable QTL (**Table 3**). The majority of the 100-seed weight QTLs in the F_2_ and F_2:3_ population showed negative additive effects with negative alleles from the parent with lower seed weight i.e., SKAF148. It is important to note that majority of the QTLs for 100 seed weight were located on chromosomes 11 and 12 suggesting the significance of these two chromosomes in regulating seed-weight inheritance.

Seed oil and protein content in soybean have a significant correlation with seed size and shape (Hacisalihoglu and Settles, 2017) as seed oil and protein content represents a major component of soybean seeds, representing 18–22% and 38–42% respectively (Wu et al., 2018). Some of the QTLs identified in this study have QTLs linkage/pleiotropy that regulate other nutritional traits. Previously, Junyi et al. (2007) and Qi et al. (2011) have identified QTLs for protein and oil content in the same region of identified QTL *qSL-11-1* on chromosome 11, which is related to seed length. QTL *qSH-9-1* on chromosome 9, regulating seed height in this study found consistent with the QTLs for oil and protein content reported by earlier workers (Csanadi et al., 2001; Priolli et al., 2015). Two QTLs *q100SW-11-2* and *q100SW-13-1* on chromosome 11 and 13 respectively regulating the weight of 100 seeds in soybean were found consistent with the QTLs for protein and oil content reported by earlier workers (Junyi et al., 2007; Qi et al., 2011). These findings indicate that seed size and shape QTLs also regulate the seed protein and oil content in soybean as soybean seeds have a major component of seed oil (18-22%) and protein content (38-42%) (Hina et al., 2020).

### 3.4 Gene ontology and candidate gene prediction within stable QTLs

Based on the mapping results, we selected seven QTLs which were stable QTLs viz., *qSL-10-1, qSW-4-1, qSV-4-1, qSV-6-1, qSLW-10-1, qSLH-10-1* and *q100SW-11-1* for gene ontology (GO) and candidate gene prediction analysis. Within the physical genomic interval of *qSL-10-1, qSW-4-1, qSV-4-1, qSV-6-1, qSLW-10-1, qSLH-10-1* and *q100SW-11-1*, the 62, 15, 13, 77, 58, 62, and 94 model genes were present, respectively that were downloaded from *SoyBase* ((http://www.soybase.org) and *EnsemblPlants* (https://plants.ensembl.org) (**Supplementary Table 1**). *Phytozome 13* was used to annotate all of the genes found in each QTL region. Each of the seven stable QTLs had a higher proportion of genes associated with the cell part, cell organelle, catalytic activity, binding, metabolic process, and cellular process which demonstrates the importance of these processes in the development of soybean seeds (Karikari et al., 2019). However, we employed PANTHER analysis, gene annotation data, and literature search to discover the potential candidate genes underlying the aforementioned seven stable QTLs accountable for soybean seed size and seed shape. A set of 66 genes out of 381 model genes inside the physical regions of the seven stable QTLs were found to be potential candidate genes influencing soybean seed size and shape based on the PANTHER analysis, gene annotation, and published literature (**Table 6**). Out of these 66 genes, eight genes each belongs to the ubiquitin-protein ligase class, oxygenase and non-receptor serine/threonine protein kinase. Seven genes each found to be associated with amino acid transporter, DNA-binding transcription factor and glycosyltransferase. Six genes had the association for ATP-binding cassette (ABC) transporter and five genes for calmodulin-related. Seven genes as a transporter and two genes were associated with aspartic protease.

**Table 6:** Sixty-six possible candidate genes predicated within seven stable QTL regions identified in this study based on PANTHER analysis, gene annotation, and available literature.

QTL	Mapped IDs	PANTHER Family	PANTHER Protein Class	References
***qSL10-1***	Glyma.10G204300	SERINE/THREONINE-PROTEIN KINASE RIO (PTHR10593)	non-receptor serine/threonine protein kinase	Unpublished^a^
	Glyma.10G204100	AMINO ACID TRANSPORTER (PTHR22950)	amino acid transporter	Unpublished^a^
	Glyma.10G204200	MYB FAMILY TRANSCRIPTION FACTOR (PTHR31003)	DNA-binding transcription factor	Unpublished^a^
	Glyma.10G203000	ATP-BINDING CASSETTE TRANSPORTER (PTHR19241)	ATP-binding cassette (ABC) transporter	Mishra et al. (2019) ^b^
	Glyma.10G203700	MAJOR FACILITATOR SUPERFAMILY DOMAIN-CONTAINING PROTEIN 10 (PTHR23504)	secondary carrier transporter	Unpublished^a^
	Glyma.10G202400	FLAVONOID 3’-MONOOXYGENASE-RELATED (PTHR24298)	oxygenase	Du et al. (2017) ^b^
	Glyma.10G202500	RING FINGER DOMAIN-CONTAINING (PTHR14155)	ubiquitin-protein ligase	Unpublished^a^
	Glyma.10G200700	EXOSTOSIN HEPARAN SULFATE GLYCOSYLTRANSFERASE -RELATED (PTHR11062)	glycosyltransferase	Unpublished^a^
	Glyma.10G205600	ATP-BINDING CASSETTE TRANSPORTER (PTHR19241)	ATP-binding cassette (ABC) transporter	Unpublished^a^
	Glyma.10G201600	AMINO ACID TRANSPORTER (PTHR22950)	amino acid transporter	Unpublished^a^
	Glyma.10G200800	FLAVONOID 3’-MONOOXYGENASE-RELATED (PTHR24298)	oxygenase	Liu et al. (2020) ^b^
***qSW4-1***	Glyma.04G135900	CHLOROPLAST ENVELOPE MEMBRANE PROTEIN-RELATED (PTHR33650)	….	Unpublished^a^
	Glyma.04G136000	ASPARTYL PROTEASES (PTHR13683)	aspartic protease	Unpublished^a^
	Glyma.04G135700	U BOX DOMAIN-CONTAINING (PTHR23315)	ubiquitin-protein ligase	Unpublished^a^
	Glyma.04G135400	TRIHELIX TRANSCRIPTION FACTOR ASIL2 (PTHR31307)	DNA-binding transcription factor	Liu et al. (2020) ^b^
	Glyma.04G136100	GLYCOGEN SYNTHASE KINASE-3 ALPHA (PTHR24057)	non-receptor serine/threonine protein kinase	Unpublished^a^
	Glyma.04G136300	EF-HAND CALCIUM-BINDING DOMAIN CONTAINING PROTEIN (PTHR10891)	calmodulin-related	Unpublished^a^
	Glyma.04G136600	EF-HAND CALCIUM-BINDING DOMAIN CONTAINING PROTEIN (PTHR10891)	calmodulin-related	Li et al. (2022) ^b^
***qSV4-1***	Glyma.04G136000	ASPARTYL PROTEASES (PTHR13683)	aspartic protease	Unpublished^a^
	Glyma.04G135700	U BOX DOMAIN-CONTAINING (PTHR23315)	ubiquitin-protein ligase	Unpublished^a^
	Glyma.04G135400	TRIHELIX TRANSCRIPTION FACTOR ASIL2 (PTHR31307)	DNA-binding transcription factor	Liu et al. (2020) ^b^
	Glyma.04G136100	GLYCOGEN SYNTHASE KINASE-3 ALPHA (PTHR24057)	non-receptor serine/threonine protein kinase	Unpublished^a^
	Glyma.04G136300	EF-HAND CALCIUM-BINDING DOMAIN CONTAINING PROTEIN (PTHR10891)	calmodulin-related	Unpublished^a^
***qSV6-1***	Glyma.06G064900	RING/FYVE/PHD ZINC FINGER DOMAIN-CONTAINING (PTHR23012)	ubiquitin-protein ligase	Unpublished^a^
	Glyma.06G066000	EF HAND DOMAIN FAMILY A1,A2-RELATED (PTHR12294)	calmodulin-related	Unpublished^a^
	Glyma.06G064700	XANTHINE-URACIL/VITAMIN C PERMEASE FAMILY MEMBER (PTHR11119)	transporter	Nasarudin (2016) ^b^
	Glyma.06G064600	TRANSCRIPTIONAL REGULATOR PROTEIN HCNGP (PTHR13464)	DNA-binding transcription factor	Unpublished^a^
	Glyma.06G064800	GLYCOGEN SYNTHASE KINASE-3 ALPHA (PTHR24057)	non-receptor serine/threonine protein kinase	Wang et al. (2018) ^b^
	Glyma.06G060700	ENHANCER OF RUDIMENTARY ERH (PTHR12373)	DNA-binding transcription factor	Unpublished^a^
	Glyma.06G063000	U BOX DOMAIN-CONTAINING (PTHR23315)	ubiquitin-protein ligase	Unpublished^a^
	Glyma.06G066500	SOLUTE CARRIER FAMILY 35, MEMBER F5 (PTHR23051)	secondary carrier transporter	Unpublished^a^
	Glyma.06G060200	IRON-SULFUR DOMAIN CONTAINING PROTEIN (PTHR21266)	oxygenase	Unpublished^a^
	Glyma.06G066700	EXOSTOSIN HEPARAN SULFATE GLYCOSYLTRANSFERASE -RELATED (PTHR11062)	glycosyltransferase	Unpublished^a^
	Glyma.06G061300	TRANSMEMBRANE 9 SUPERFAMILY PROTEIN (PTHR10766)	transporter	Unpublished^a^
***qSLW10-1***	Glyma.10G203600	MAJOR FACILITATOR SUPERFAMILY DOMAIN-CONTAINING PROTEIN 10 (PTHR23504)	secondary carrier transporter	Unpublished^a^
	Glyma.10G202500	RING FINGER DOMAIN-CONTAINING (PTHR14155)	ubiquitin-protein ligase	Unpublished^a^
	Glyma.10G203500	FLAVONOID 3’-MONOOXYGENASE-RELATED (PTHR24298)	oxygenase	Whaley et al. (2015) ^b^
	Glyma.10G203000	ATP-BINDING CASSETTE TRANSPORTER (PTHR19241)	ATP-binding cassette (ABC) transporter	Unpublished^a^
	Glyma.10G204200	MYB FAMILY TRANSCRIPTION FACTOR (PTHR31003)	DNA-binding transcription factor	Unpublished^a^
	Glyma.10G204100	AMINO ACID TRANSPORTER (PTHR22950)	amino acid transporter	Unpublished^a^
	Glyma.10G200000	GLYCOSYLTRANSFERASE (PTHR12526)	glycosyltransferase	Unpublished^a^
	Glyma.10G205600	ATP-BINDING CASSETTE TRANSPORTER (PTHR19241)	ATP-binding cassette (ABC) transporter	Unpublished^a^
	Glyma.10G201600	AMINO ACID TRANSPORTER (PTHR22950)	amino acid transporter	Unpublished^a^
	Glyma.10G206600	SERINE/THREONINE-PROTEIN KINASE RIO (PTHR10593)	non-receptor serine/threonine protein kinase	Dasmandal et al. (2020) ^b^
	Glyma.10G200800	FLAVONOID 3’-MONOOXYGENASE-RELATED (PTHR24298)	oxygenase	Liu et al. (2020)
***qSLH10-1***	Glyma.10G203700	MAJOR FACILITATOR SUPERFAMILY DOMAIN-CONTAINING PROTEIN 10 (PTHR23504)	secondary carrier transporter	Unpublished^a^
	Glyma.10G203000	ATP-BINDING CASSETTE TRANSPORTER (PTHR19241)	ATP-binding cassette (ABC) transporter	Mishra et al. (2019) ^b^
	Glyma.10G203500	FLAVONOID 3’-MONOOXYGENASE-RELATED (PTHR24298)	oxygenase	Whaley et al. (2015) ^b^
	Glyma.10G204100	AMINO ACID TRANSPORTER (PTHR22950)	amino acid transporter	Unpublished^a^
	Glyma.10G202500	RING FINGER DOMAIN-CONTAINING (PTHR14155)	ubiquitin-protein ligase	Unpublished^a^
	Glyma.10G204300	SERINE/THREONINE-PROTEIN KINASE RIO (PTHR10593)	non-receptor serine/threonine protein kinase	Unpublished^a^
	Glyma.10G200700	EXOSTOSIN HEPARAN SULFATE GLYCOSYLTRANSFERASE -RELATED (PTHR11062)	glycosyltransferase	Unpublished^a^
	Glyma.10G205600	ATP-BINDING CASSETTE TRANSPORTER (PTHR19241)	ATP-binding cassette (ABC) transporter	Unpublished^a^
	Glyma.10G201600	AMINO ACID TRANSPORTER (PTHR22950)	amino acid transporter	Unpublished^a^
***q100SW11-1***	Glyma.11G055700	MONOOXYGENASE (PTHR13789)	oxygenase	Maruyama et al. (2020) ^b^
	Glyma.11G056500	EF-HAND CALCIUM-BINDING DOMAIN CONTAINING PROTEIN (PTHR10891)	calmodulin-related	Unpublished^a^
	Glyma.11G054400	MITOGEN-ACTIVATED KINASE KINASE KINASE (PTHR24361)	non-receptor serine/threonine protein kinase	Unpublished^a^
	Glyma.11G054700	NICOTINATE PHOSPHORIBOSYLTRANSFERASE (PTHR11098)	glycosyltransferase	Unpublished^a^
	Glyma.11G055100	MITOGEN-ACTIVATED KINASE KINASE KINASE (PTHR24361)	non-receptor serine/threonine protein kinase	Unpublished^a^
	Glyma.11G250000	HISTONE-LIKE TRANSCRIPTION FACTOR CCAAT-RELATED (PTHR10252)	DNA-binding transcription factor	Unpublished^a^
	Glyma.11G245300	OXA1 (PTHR12428)	transporter	Unpublished^a^
	Glyma.11G246300	E3 UBIQUITIN-PROTEIN LIGASE DMA1-RELATED (PTHR15067:SF7)	ubiquitin-protein ligase	Unpublished^a^
	Glyma.11G247800	AMINO ACID TRANSPORTER (PTHR22950)	amino acid transporter	Unpublished^a^
	Glyma.11G250200	FLAVONOID 3’-MONOOXYGENASE-RELATED (PTHR24298)	oxygenase	Hale et al. (2020) ^b^
	Glyma.11G243000	BETA-1,4-MANNOSYL-GLYCOPROTEIN BETA-1,4-N-ACETYLGLUCOSAMINYL-TRANSFERASE (PTHR12224)	glycosyltransferase	Unpublished^a^
	Glyma.11G254500	EXOSTOSIN HEPARAN SULFATE GLYCOSYLTRANSFERASE -RELATED (PTHR11062)	glycosyltransferase	Unpublished^a^

a The reference related with this gene are not publish, but submitted in GenBank.

b This gene was completely matched with published gene.

PANTHER revealed that the Glyma.04G135900 gene did not encode any proteins.

## 4 Discussion

The economically important traits that affect soybean production and quality include seed size and shape (Gandhi, 2009). Therefore, creating soybean cultivars with better seed sizes and shapes is thought to be a crucial goal of soybean breeding projects. To create better cultivars, it is necessary to have a detailed understanding of genetic architecture and the process behind the trait of interest. Seed size and shape are intricate quantitative features that are controlled by numerous genes and are extremely sensitive to their environment (Hina et al., 2020). Due to small-sized mapping populations and low-density genetic maps, many QTLs related to soybean seed size and shape have been reported over the past few decades but have not yet been stable and confirmed (Zhang et al., 2004; Niu et al., 2013; Kato et al., 2014; Wu et al., 2018). As a result, they cannot be inferred for breeding improved seed sizes and shapes in soybean. Therefore, the current study used F_2_ and F_2:3_ mapping populations derived from the cross of vegetable (AGS 457) and seed type (SKAF 148) soybean, evaluated in the 2020 and 2021 growing seasons at the Indian Agricultural Research Institute, New Delhi, India in order to find stable significant QTLs and potential candidate genes for soybean seed size and shape traits.

### 4.1 Phenotypic analysis of seed shape and seed weight

An essential economic trait influencing soybean yield is seed shape and seed weight. Therefore, the breeders have been pursuing to develop varieties with desirable seed shapes and seed weights with an eventual higher yield. However, like yield, seed shape and seed weight are also polygenic traits controlled by numerous genes and hence hard to manage effectively through conventional approaches. Despite the fact that many QTLs relating to soybean seed weight, size, and shape have been reported over the past few decades, most of these QTLs remained unutilized owing to their unstable or unconfirmed performances in other genetic backgrounds. Therefore, the current study targeted discovering QTLs in one generation of the population (F_2_) and confirm it in another generation (F_2:3_) for reliability and applicability of the QTLs in the breeding program. Genetic diversity between the parental genotypes is essential for mapping QTL, the wider the better. The genotypes used in this study i.e., AGS457 and SKAF148 differed significantly for the seed shape and seed weight traits. It caused the mapping populations to become extremely variable, which allowed for the mapping of multiple novel QTLs.

Additionally, the variability in the F_2_ population facilitated recombination among the alleles resulting in the recovery of transgressive segregants. Li et al. (2008) and Zhang et al. (2010) also reported the appearance of transgressive segregants in segregating populations of soybean. The ability to select one trait through another is provided by the correlation among the target traits. In this study, a strong association was found between the characteristics linked to seed shape and seed weight. The wider range and higher value of the correlation coefficients (-0.05 to 0.75) indicated their strength of association. A substantial positive association was found between seed length and seed width, seed volume, seed length to width ratio, seed length to height ratio, seed width to height ratio, and seed weight (Li et al., 2020). Similarly, seed width had a strong positive relationship with seed length, height, volume, and weight, while there was no statistically significant relationship between seed height and 100 seed weight which is consistent with those in Xie et al. (2014). These findings demonstrated the significance of seed length and width in defining the shape of the soybean seed and overall yield.

### 4.2 Genetic control of seed shape and seed weight

Seed weight and seed shape are complex traits and a host of loci are involved in genetic control of them (Liang et al., 2016; Khosla et al., 2020). The normal distribution of the traits in the segregating generations and the number of QTLs mapped for it support the concept of multi-genic control of these traits. In this study, 42 QTLs for seed shape and seed weight-related traits were mapped in F_2_ (17 QTLs) and F_2:3_ (25 QTLs) populations. Seven out of the 42 QTLs were mapped in both generations and hence can be regarded as stable QTLs. Similarly, 13 of the 42 QTLs detected in the current study matched with the previously reported QTLs, while the remaining 29 were reported for the first time i.e., novel. Out of these 29 novel QTLs, 17 appeared to be major QTLs with PVE of more than 10%. Out of the five QTLs for seed length mapped here, one QTL i.e., *qSL-10-1* was a major effect and stable QTL (13.7% PVE) and would be suitable for deployment in the breeding program. Similarly, *qSW-4-1* was a stable QTL for seed width. The *qSW-4-1* along with three other QTLs of seed width viz., *qSW-2-1, qSW-2-2*, and *qSW-6-1* corresponded to similar QTLs reported by Salas et al. (2006); Xu et al. (2011); Niu et al. (2013) and Yu et al. (2018). The QTLs *qSW-6-1* and *qSW-2-1* had PVE 17.56% and 17.09%, respectively and fit to deploy for improvement of seed width. Similarly, two QTLs for seed height viz., *qSH-4-1* and *qSH-6-1* correspond to the similar QTLs reported by Niu et al. (2013) and Yu et al. (2018) respectively. However, QTL *qSH-9-1* accounted for 17.28% of phenotypic variance and was a novel QTL for seed height.

For seed volume, six novel QTLs were mapped on chromosomes 2, 4, 6, and 10, of which, QTL *qSV-4-1* with PVE 33.94% was a major one and fit for deployment in the breeding program. The parental genotypes, AGS457 and SKAF had seed volumes of 412.42 mm^3^ and 127.31 mm^3^, respectively. This huge difference in seed volume in the parental genotypes supported the discovery of multiple novel QTLs for seed volume. The ratio of seed length to width (SLW) is crucial in determining the shape of the seed. One out of the four QTLs for SLW i.e., *qSLW-10-1* was located in the marker region Satt592-Sat_341 and accounted for 19.6% of PVE. The positive alleles from the genotype AGS 457 contributed to the positive additive effect of this QTL. The seed width-to-height ratio (SWH) and seed length-to-height ratio (SLH) had four QTLs each. Two of the four QTLs for seed width to height ratio i.e., *qSWH-4-1* and *qSWH-6-1* were novel and major and suitable for deployment in the breeding program. The remaining QTLs for SLH and SWH are the same as reported earlier by Yang et al. (2013) and Rathod et al. (2019). Seed size is an important trait for adaptation to a certain environment. It also determines the overall yield of soybean (Tao et al., 2017). In this study, 11 QTLs were mapped for seed size i.e., 100-seed weight, out of which eight were novel and the rest corresponded to those reported earlier by Xu et al. (2011), Niu et al. (2013) and Liu et al. (2018). The huge difference in seed size of the parental genotypes contributed towards the mapping of several QTLs for seed size. There was one stable QTL for seed size i.e., *q100SW-11-1*, and deserves further confirmation and deployment. Some of the seed size and shape QTLs viz *qSL-11-1, qSH-9-1, q100SW-11-2*, qSLH-5-1, *qSWH-11-1, qSWH-13-1* and *q100SW-13-1* were found consistent with the QTLs for oil and protein content in soybean reported by earlier workers (Csanadi et al., 2001; Junyi et al., 2007; Qi et al., 2011; Priolli et al., 2015). These findings indicate that these seed size and shape QTLs regions showed QTLs linkage/pleiotropy which regulates other nutritional traits viz. seed protein and oil content in soybean (Hina et al., 2020).

### 4.3 Candidate gene analysis for seed shape and 100 seed-weight

Identification of the actual candidate gene that lies beneath the QTL region is crucial for improving the target trait through through breeding approach. In this study, using information from the available literature, gene annotation, and bioinformatics tools, potential candidate genes for soybean seed shape and 100-seed weight were identified. The seven stable QTLs identified in this study viz., *qSL-10-1, qSW-4-1, qSV-4-1, qSV-6-1, qSLW-10-1, qSLH-10-1*, and *q100SW-11-1* were used for this purpose. Out of the 381 model genes extracted from the physical genomic interval of the seven stable QTLs, 66 were considered as potential candidate genes as per PANTHER analysis, gene function, and available literature (Karikari et al., 2019). The candidate genes are primarily associated with cell components, catalytic activity, transportation, metabolic, and cellular processes, all of which are crucial for seed development (Fan et al., 2006; Li and Li, 2014). For instance, the oxygenase protein class includes the genes Glyma.10G202400, Glyma.10G200800, Glyma.06G060200, Glyma.10G203500, and Glyma.11G055700, which are associated with the QTLs *qSL-10-1, qSV-6-1, qSLW-10-1*, *qSLH-10-1* and *q100SW-11-1*. These genes affect soybean seed size (Zhao et al., 2016). Similar to this, members of the protein family E3 ubiquitin-protein ligase are involved in the ubiquitin-proteasome pathway. The E3 ubiquitin-protein ligase genes Glyma.10G202500, Glyma.04G135700, Glyma.06G064900, and Glyma.06G063000, were identified here as potential candidate genes. Members of this protein family include genes for GW2 in rice (Choi et al., 2018), TaGW2 in wheat (Lv et al., 2022), and ZmGW2 in maize (Kong et al., 2014), all of which have been reported to have a significant impact on seed development (Ge et al., 2016; Lv et al., 2022).

The link between the source (leaf) and sink (seed) regulates seed development in plants (Snyder, 1993). Therefore, the genes Glyma.10G204100, Glyma.10G201600, Glyma.11G247800, Glyma.10G203000, Glyma.10G205600 and Glyma.06G064700 from the amino acid transporter and ATP-binding cassette (ABC) transporter are plausible candidate genes for seed shape and seed weight in soybean (Li et al., 2019). Since a calmodulin-like domain protein kinase is necessary for storage product accumulation during seed development in rice (Asano et al., 2002), genes Glyma.04G136300, Glyma.04G136600 Glyma.06G066000 and Glyma.11G056500 were suspected of being involved in soybean seed development. Serine/threonine protein kinase is involved in ABA signaling, and is crucial for the regulation of seed growth and dormancy. Therefore, the non-receptor serine/threonine protein kinase genes Glyma.10G204300, Glyma.04G136100, Glyma.06G064800, Glyma.10G206600, Glyma.11G054400, and Glyma.11G055100 can be potential candidate genes influencing soybean seed development. Candidate genes discovered in this study are involved directly or indirectly in regulating seed development, as well as seed size and shape, such as cell component, storage of proteins and lipids, transport, metabolic process, signal transduction of plant hormones, degradation of the ubiquitin-proteasome pathway, and fatty acid beta-oxidation (**Table 6**). Hence, based on the gene function, GO, and literature search, the above 66 genes were considered as the most potentially possible candidate genes for regulating the seed sizes and shapes in soybeans. With the help of these findings, strategies for increasing soybean yield can be developed by comprehending functional networks. Important genetic resources for soybean are made available by the markers and candidate genes discovered in this study. Lastly, the major and stable QTLs identified in the present study ought to be mapped finely for the identification of tightly linked markers for effective molecular breeding towards improving seed shape, seed weight, and yield of soybean.

## 5 Conclusion

The present study used vegetable and seed soybean-derived F_2_ and F_2:3_ mapping populations to detect QTLs as well as mine possible candidate genes controlling seed shape and 100-seed weight in soybean. This study has identified a total of 42 QTLs for seed shape and 100 seed weight out of which 29 were novel. In addition, seven out of 42 QTLs were stable QTLs identified in both F_2_ and F_2:3_ mapping populations and five of them were major ones viz., *qSL-10-1, qSW-4-1, qSV-4-1, qSLW-10-1* and *qSLH-10-1*. In total, 66 possible candidate genes were mined within the seven stable QTLs and most of them belonged to ubiquitin-protein ligase and oxygenase that have been earlier reported to play significant roles in seed/organ size development and regulation. Our study provides the major and stable QTLs and candidate genes regulating seed shape and 100 seed weight in soybean, and these findings will be of great use for marker-assisted breeding (MAB) of soybean varieties with improved seed-weight and desired seed shape.

## Data availability statement

The original contributions presented in the study are included in the article/**Supplementary Material**. Further inquiries can be directed to the corresponding author.

## Author contributions

RK, RP, KG, SL and AT conceived and designed the experiments. MS, MT, PD, RM, AyR, DS and AmR assisted the experiments. RK, MS and AT analyzed the data. RK and AT drafted and revised the manuscript. All authors have read and agreed to the current version of the manuscript.
